# Post-stroke experiences and health information needs among Chinese elderly ischemic stroke survivors in the internet environment: a qualitative study

**DOI:** 10.3389/fpsyg.2023.1150369

**Published:** 2023-08-16

**Authors:** Yufan Hu, Xichenhui Qiu, Cuiling Ji, Fang Wang, Manlan He, Lei He, Lu Chen

**Affiliations:** ^1^Department of Neurosurgery, Nanjing Drum Tower Hospital Clinical College of Nanjing University of Chinese Medicine, Nanjing, Jiangsu, China; ^2^Department of Neurosurgery, Nanjing Drum Tower Hospital, The Affiliated Hospital of Nanjing University Medical School, Nanjing, Jiangsu, China; ^3^Health Science Center, Shenzhen University, Shenzhen, China

**Keywords:** elderly patient, stroke, health information-seeking behavior, qualitative study, ischemic stroke

## Abstract

**Background:**

Elderly stroke survivors are encouraged to receive appropriate health information to prevent recurrences. After discharge, older patients seek health information in everyday contexts, examining aspects that facilitate or impair healthy behavior.

**Objectives:**

To explore the experiences of older stroke patients when searching for health information, focusing on search methods, identification of health information, and difficulties faced during the search process.

**Methods:**

Using the qualitative descriptive methodology, semi-structured interviews were conducted with fifteen participants.

**Results:**

Participants associated the health information they sought with concerns about future life prospects triggered by perceived intrusive changes in their living conditions. Based on the participants’ descriptions, four themes were refined: participants’ motivation to engage in health information acquisition behavior, basic patterns of health information search, source preferences for health information, and difficulties and obstacles in health information search, and two search motivation subthemes, two search pattern subthemes, four search pathway subthemes, and four search difficulty subthemes were further refined.

**Conclusion:**

Older stroke patients face significant challenges in searching for health information online. Healthcare professionals should assess survivors’ health information-seeking skills, develop training programs, provide multichannel online access to health resources, and promote secondary prevention for patients by improving survivors’ health behaviors and self-efficacy.

## 1. Introduction

Stroke is a leading cause of mortality and disability worldwide, causing more than 143 million disability-adjusted life years lost yearly (Feigin et al., 2022). Ischemic stroke represents about 70% of the total, with the primary incidence in older adults (Bambhroliya et al., 2018). Patients are usually discharged home without follow-up care after the acute phase; nevertheless, the disease causes significant disruption in survivors’ lives. In the long run, they may have a reduced quality of life due to a lack of disease-related health information, inadequate professional rehabilitation, and low levels of social engagement (Chuluunbaatar et al., 2016; Crichton et al., 2016). These factors might lead to adverse health outcomes such as stroke recurrence and mood disorders (Bi and Wang, 2022; Chau et al., 2022; Wang et al., 2022). Therefore, post-acute stroke survivors must have access to proper disease knowledge and specialized rehabilitation information to support their physical and psychological recovery and promote positive health outcomes.

Evidence shows that 71.2% of Chinese users are accustomed to using the internet to access medical services (Liu and Yang, 2022). However, only 14.3% of stroke patients with high school or higher education level access disease health information online (Zhao et al., 2022). This information includes regular physical exercise and maintaining a healthy lifestyle, which can reduce the effects of stroke sequelae and disease and improve long-term health status (Kramer et al., 2019; Hou et al., 2021). However, health information services for older patients are often inadequate to fully address their ongoing health literacy needs after the acute phase, including recovery of functional independence, quality of life, and social participation (Avan et al., 2019; Guo et al., 2021). Older stroke patients rated the transition from the hospital to the home setting as poor. They felt abandoned and dissatisfied with existing inpatient services due to a perceived lack of professional attention to their rehabilitation needs and goals (Pucciarelli et al., 2017; Gödde et al., 2022). These findings suggest the need for personalized and targeted disease health information services to meet older stroke survivors’ ongoing physical and mental health needs.

The purpose of the present study is to explore the experiences and needs of older ischemic stroke patients in the online environment to access health information about their disease, with a focus on perceived health, reflections on their health behaviors, and how this is related to their overall experience of resuming daily life concerning the potential sequelae of stroke. This knowledge might help identify supportive strategies for providing health behavior counseling in clinical settings and follow-up care.

## 2. Materials and methods

### 2.1. Design

A descriptive qualitative study with semi-structured interviews was carried out. This study design was chosen because it is grounded in the principles of naturalistic inquiry (Colorafi and Evans, 2016).

### 2.2. Sample selection and recruitment

Elderly ischemic stroke survivors were voluntarily recruited from the Departments of Neurosurgery and Neurology at Drum Tower Hospital, Nanjing University School of Medicine, China. Purposive sampling methods were used to ensure a saturated sample size and to generate a broad participant response. Recruitment posters were displayed in each ward of the Departments of Neurosurgery and Neurology at the participating hospitals. Case managers registered patients interested in participating in the program. After contacting and informing them of the study, case managers sent potential participants’ names and bed numbers to a master’s student on the project team to confirm their eligibility. Eligible participants signed a consent form before the start of the interview.

### 2.3. Participant eligibility

Participants must be (1) 60 years of age or older, (2) have been diagnosed with an ischemic stroke for at least 1 month, (3) be alert, have a GCS score of 15, (4) be able to communicate in Chinese, (5) have an education level of junior high school and above, and (6) be have searched for health-related information on the Internet at least once in the past month. Survivors who lost the ability to communicate due to aphasia were excluded. The sample size for this study was determined based on the principle of “information saturation,” i.e., patients were saturated with interview data, and no new themes or sub-themes emerged during the interviews.

### 2.4. Data collection

The literature was searched, and a subject matter expert with qualified research experience created a preliminary interview outline. We selected the top three two cases from the patients who met the selection criteria for pilot interview testing. We performed personal in-depth interviews to generate rich data and consensus themes about the experiences of older stroke survivors searching for online health information. Changes were made to the interview outlines that were challenging to understand, resulting in a formalized interview outline (Table 1). The interview focused on stroke survivors’ experiences when searching for health information.

**Table 1:** Questions in the semi-structured interview guide.

1. Will the internet often pay attention to disease and health-related information?
2. How to obtain health information about stroke?
3. What are the main network channels to find stroke health information? Which is the most commonly used? Why?
4. What health information has been retrieved about stroke?
5. How do you view the communication groups of patients and doctors, and nurses based on social media (such as WeChat, QQ, etc.)?
6. How does the health information obtained from network resources affect you?
7. How do you feel about the quality of health information about various diseases online
8. How do you judge the authenticity of these diseases and health information?
9. What difficulties do you have in obtaining disease-related health information? What kind of help do you want?
10. If there were an online support platform for stroke patients, what would you like it to be? (In what form, functions, aspects of information, information providers…)
11. Is there anything else you would like to say on today’s topic?

Patients were selected for face-to-face interviews at the end of treatment in a quiet classroom on the unit. Data were collected using semi-structured interviews and observation methods by two nurses (MH and LH, both supervising nurses with extensive working experience in stroke care) who received specialized training in qualitative research methods before the interviews to ensure rigor in the data collection process. The researcher introduced herself before the interview, explained the study’s purpose and methodology, and provided informed written consent obtaining. The timing of the interview was determined according to the situation. All interviews were audio-recorded, and the researcher paid attention to non-verbal characteristics, including facial expressions, movement, and speed of speech during the interview and kept a record of the interview. The interviews were stopped when nothing new appeared in the interview results, i.e., data saturation, and privacy was protected by numbering.

### 2.5. Data analysis

The audio recordings were transcribed separately by two researchers within 24 h of the interviews, checked for accuracy against the original transcripts, and any inconsistencies were resolved. Two researchers also systematically analyzed the data using framework analysis and following the seven stages of the framework approach in the analysis process. This included (1) transcription, (2) familiarization interview, (3) coding, (4) development of a working analytic framework using a coding scheme (Gale et al., 2013) (for the final themes versus subthemes generated, (5) application of the analytic framework to the data, (6) charting of the data, and (7) data interpretation. Nvivo software was used for data management). A third researcher’s help was sought when opinions created disagreements. Results were reviewed and confirmed by all authors.

**Table 2:** Characteristics of the study sample (*N* = 15).

Number	Gender	Age (years)	Occupation	Monthly personal income (CNY)	Marital status	Educational level	Residence	Medical insurance	Number of strokes	Year of internet access	Daily internet time	Frequency of active seeking of health information
P1	Female	60	Freelance	<1,000	Married	senior secondary	Couples living together	Have	1	1-3 years	≥3 h	1–3/month
P2	Male	70	Retirement	1,000–3,000	Married	Secondary	Couples living together	Have	≥3	1-3 years	1–3 h	1–3/month
P3	Male	61	Staff	>3,000	Married	University and above	Couples living together	Have	2	≥3 years	<1 h	1–3/month
P4	Male	62	Staff	>3,000	Widowed	senior secondary	Solitary	Have	1	≥3 years	1–3 h	≥4 month
P5	Male	63	Farmers	<1,000	Married	Secondary	Couples living together	No	1	<1 year	<1 h	1–3/month
P6	Female	64	Freelance	1,000-3,000	Widowed	Secondary	Children living together	Have	2	≥3 years	≥3 h	≥4 month
P7	Female	71	Retirement	>3,000	Married	Secondary	Couples living together	Have	2	≥3 years	≥3 h	1–3/month
P8	Male	63	Construction worker	1,000–3,000	Married	Secondary	Couples living together	Have	1	1-3 years	<1 h	≥4 month
P9	Female	64	Staff	1,000–3,000	Married	Senior secondary	Couples living together	Have	≥3	≥3 years	<1 h	≥4 month
P10	Male	73	Retirement	>3,000	Married	University and above	Couples living together	Have	1	≥3 years	≥3 h	1–3/month
P11	Female	72	Unemployed	<1,000	Married	Secondary	Couples living together	Have	1	<1 year	≥3 h	≥4 month
P12	Male	63	Freelance	<1,000	Married	Secondary	Couples living together	Have	1	<1 year	1–3 h	1–3/month
P13	Male	61	Farmers	1,000–3,000	Married	Secondary	Couples living together	No	1	≥3 years	1–3 h	1–3/month
P14	Female	60	Retirement	1,000–3,000	Married	senior secondary	Children living together	Have	≥3	1-3 years	≥3 h	1–3/month
P15	Female	63	Farmers	<1,000	Married	senior secondary	Couples living together	Have	1	<1 year	<1 h	1–3/month

### 2.6. Data reliability

Lincoln and Guba (1985) used the four principles of credibility to ensure data reliability. Credibility is established through ongoing observations and regular meetings between research team members to discuss data interpretation and reach a consensus on new findings. To ensure transferability, we obtained a detailed description of the participant’s experience of accessing health information after stroke, including contextual factors and direct quotes from the participant.

### 2.7. Ethical considerations

The Investigation and Behavioral Research Ethics Committee of Nanjing Drum Tower Hospital, the Affiliated Hospital of Nanjing University Medical School (2022-692). Following the Declaration of Helsinki, the rights and security of the participants were guaranteed. Study objectives and procedures were explained to participants during recruitment, and written informed consent was obtained. Participants were told they had the right to refuse to participate or withdraw from the study at any time. All data were anonymous and stored securely in encrypted files. Only members of the research team had access to the data.

**Table 3:** Summary of themes emerging from the data.

Main themes	Sub-themes
1. Health information search motivation	1.1 Physical and psychosocial recovery
	1.2 Home guidance from professionals
2. Basic model of health information search	2.1 active health information-seeking behavior
	2.2 Proactive health information-seeking behavior
3. Source preferences for health information	3.1 Search engines
	3.2 WeChat
	3.3 short videos
	3.4 online medical platforms and health apps
4. Difficulties and obstacles in health information search	4.1 Physiological degeneration
	4.2 Lack of information-seeking skills
	4.3 Children played a weaker role
	4.4 Disease and health information service system needs to be improved

## 3. Results

The study consisted of interviews with 15 older adult ischemic stroke survivors. The mean age was 64.67 years (standard deviation: 4.32 years), with slightly more men (53%). Most participants were employed before the stroke (67%), while all were either unemployed or retired after the stroke. Most participants (93%) lived with their family or caregivers. Most participants (60%) experienced only one stroke. Half (47%) reported using the internet for three years or more, and most (67%) reported using the internet for one hour or more daily. More details about the participants’ characteristics can be found in Table 2.

Four themes and twelve sub-themes emerged from the data and are summarized in Table 3.

### 3.1. Theme 1: health information search motivation

#### 3.1.1. Sub-Theme 1.1 physical and psychosocial recovery

Since most participants were in the process of recovery and felt their responsibilities reversed (i.e., from providers or caregivers who were able to function independently to dependent caregivers), they expressed a strong desire to return to functioning that would allow them to resume self-management, including being able to cook for themselves, becoming less dependent on others, and resuming previous activities.

*“I am devastated when my family at times does not get what I say. On top of that, I could not move and needed my family to take care of me… It’s a big shock.” (P4 and p9).*

*“I hope that one more day when I can cook for myself without the help of my children” (P6).*

Participants emphasized the importance of peer support, and they found it fruitful to interact with fellow patients, as they could exchange first-hand knowledge of stroke and recovery and seek motivation and psychological support from each other.

*“I felt very little joy when I was sick, and whenever a problem would arise, I would become very anxious because I did not know what to do.” (P11).*

*“After gradually learning about the disease, I have begun to meet more other survivors on the internet (*e.g.*, Douyin, WeChat). I talk to them about stroke-related things…” (P14).*

#### 3.1.2. Sub-theme 1.2 home guidance from professionals

Regarding rehabilitation after discharge, participants hoped to receive direct guidance from professionals at home. Without the relevant guidance and guidance, participants felt lost and unsure which rehabilitation program would help their recovery.

*“When we got out of the hospital, we wanted to be able to very easily find professional knowledge about rehabilitation because we did not know much about it. They are very professional and would be able to give us lessons on how to rehabilitate.” (P1).*

*“The guidance and advice provided by rehabilitation specialists are important, and we will follow them… After we are discharged from the hospital, it will be better if the professional applets or rehabilitation videos can help us to do home rehabilitation instead of doing it by ourselves by memory, which will improve the efficiency of our rehabilitation.” (P5).*

As some participants often had to travel to more distant hospitals for rehabilitation or review, they felt fatigued with the amount of commuting and reported finding primary care physicians in internet-based hospitals for rehabilitation strategy consultations.

*“I often had to travel alone for three hours by car to another city for a review.” (P10).*

*“When I have any questions or discomfort, I can always find the right professional for consultation via the internet.” (P8).*

### 3.2. Theme 2: the basic model of health information search

#### 3.2.1. Sub-theme 2.1 active health information-seeking behavior

Active health information-seeking behavior is characterized by the patient’s proactive actions and problem-solving when a health problem arises to prevent it from worsening or getting out of control.

*“Any discomfort I have I go to the internet and search for things like can I still walk on my legs? Will the stroke come back?” (P3 and P5).*

*“At first, when I could not move my left arm, I was afraid that my other arm would also be affected, so I would often search the internet for such cases” (P1).*

#### 3.2.2. Sub-theme 2.2 proactive health information-seeking behavior

Proactive health information-seeking behavior is characterized by the patient’s consciousness of taking preventive measures before a health problem arises and taking full advantage of their initiative to prevent it. Overall, the timing of proactive health information-seeking behavior is higher than that of proactive health information-seeking behavior. Proactive health information-seeking behavior is also higher than active health information-seeking behavior.

*“I was worried that I would have a relapse, so I searched the internet a lot on how to prevent relapse?” (P4).*

*“I heard from my companion that this is possible to pass on to offspring, so I will search how to avoid stroke?” (P14).*

Due to differences in health status and lifestyle, the emphasis on proactive and anticipatory modes of health information-seeking may vary among older adults (one primary and one secondary, or equivalent). Whether proactive or prospective-active, health information-seeking is proactive, health information-seeking is proactive, and purposeful health information-seeking is active, purposeful health information acquisition.

### 3.3. Theme 3: source preferences for health information

#### 3.3.1. Sub-theme 3.1 search engines

The proportion of search engines used was the highest. Patients said that search engines could search all kinds of health information conveniently and timely and comprehensively present all kinds of search results; therefore, they try to search on search engines when they have any discomfort.

*“Whatever it is, I will habitually use search engines (Baidu to search, such as looking for hospitals and doctors)” (P9).*

*“The information on the search engine (Baidu) is more comprehensive, not only text but also pictures and videos.” (P15).*

Participants also expressed that, due to the open nature of the internet, sufficient attention should be paid to the quality and feasibility of the health information obtained on various diseases.

*“The first few messages on the search engine (Baidu) are always false advertisements of some hospitals, and sometimes it is difficult to distinguish.” (P15).*

#### 3.3.2. Sub-theme 3.2 weChat

As the social software with the broadest coverage, WeChat plays an essential role in health information search. Most older adults do not have a high-quality education, and among some of the elderly patients interviewed, WeChat was the first app they used to access the internet and even the only way to obtain electronic health information.

*“In the department’s WeChat patient group, I can learn about stroke diseases, such as what a stroke is. What should I pay attention to in my diet after being discharged from the hospital? (P2).*

*“WeChat patient groups have peers who share their experiences of rehabilitation and care, and these experiences can relieve my anxiety.” (P7).*

#### 3.3.3. Sub-theme 3.3 short videos

Short videos to find information materials are emerging. Short videos based on mobile networks are also becoming a source of health information. Patients stated that compared to other search methods, short videos require a relatively low level of education; they are more tolerant of physiological conditions and more flexible in their operation.

*“As I get older, I know fewer words and rely on my ears to listen to videos most of the time.” (P11).*

*“Since my left arm is difficult to move, it is difficult to type with both hands, and I can only rely on my right limb to slide up and down the short video.” (P1).*

#### 3.3.4. Sub-theme 3.4 online medical platforms and health apps

Online medical platforms and health apps were used least frequently. The analysis of the interview data may be demanding in terms of personal cognition and electronic literacy.

*“Although I downloaded the health app, there were some features that I could not fully understand, and I could not use it skillfully.” (P4).*

*“I like to watch some science videos on the health app to pass the time.” (P3).*

### 3.4. Theme 4: difficulties and obstacles in health information search

#### 3.4.1. Sub-theme 4.1 physiological degeneration

Most expressed that physiological deterioration was a natural consequence of human aging, and although it was challenging to overcome, they all said they accepted it.

*“My eyesight has been deteriorating, and I have been able to look at my phone for at maximum one hour.” (P3).*

*“After studying the rehabilitation videos posted by the rehabilitation doctor in the WeChat group, I invariably forget them quickly, and my recollection is worse than previously” (P7).*

#### 3.4.2. Sub-theme 4.2 lack of information-seeking skills

Survivors recognized the value of good information-seeking skills and noted that this helped improve their search results.

*“Good search skills help. I think it helps me find the information I need faster.” (P6, P10, and P13).*

*“[After learning information-seeking skills] now I can distinguish advertising information in search engines. In the beginning, I always clicked into the advertising information.” (P15).*

However, despite the recognized value of good information-seeking skills, some older participants adopted a more passive attitude toward information-seeking for disease knowledge and did not need additional learning.

*“I think at this age, if I can eat well, live well and not starve, that’s enough.” (P7 and P10).*

#### 3.4.3. Sub-theme 4.3 children played a weaker role

Regarding the type of people for social support, children were the most desired caregiver group reported by participants. Nevertheless, patients were less likely to mention how their children helped them access health information independently. This may be related to China’s social environment, demographics, and family relationships.

*“Children are required to work and can only return once a week…” (P3).*

*“The kids work abroad and can only come home during holidays, and they hired caregivers to take care of me.” (P4, P9, and P10).*

#### 3.4.4. Sub-theme 4.4 disease and health information service system needs to be improved

While some participants could search for health information using the disease health services system, we identified a gap between the needs of stroke survivors and the system’s design.

*“I was unable to make representations of medical terminology because I normally use spoken language, and they were difficult to convert into valid search statements.” (P9).*

Other participants noted that features of their health information search site, such as the site’s font being too small and the color of links not being prominent, were barriers to efficiently searching for health information.

*“When I was healthy, my hands could click on any link in the information interface at will, but now it is hard to control my hands on my own…” (P12 and P14).*

## 4. Discussion

This study provided vivid evidence about the health information-seeking experiences of older Chinese stroke survivors during the acute and recovery periods. Effectively searching for accurate health information from a large amount of health information is a complex process that involves multiple interrelated aspects, such as physical, psychological, and social care domains. Often, these aspects are intertwined, as reported in prior studies.

This study found that survivors bear significant physical, psychological, and financial burdens during rehabilitation from stroke. Most patients reported muscle pain, weakness, and numbness that reduced flexibility in using online devices, consistent with the findings of Kalavina et al. (2019). These findings suggest that healthcare professionals must refine rehabilitation management programs for individual survivors to address specific rehabilitation needs effectively. The evidence suggests that health information education developed after assessing survivor goals is more effective than traditional health education (Liu et al., 2021). The present study showed that because they usually have a sudden stroke onset and are not prepared for the change in their role from family laborer to family caretaker (Chattopadhyay et al., 2017; Chen et al., 2021), their neurological and endocrine systems are strongly stimulated in adapting to the “new normal” experience of daily life, resulting in great psychological distress, such as feelings of guilt, social isolation, anxiety, and depression (Ma et al., 2022), decreasing their motivation to search for health information. Adequate education and support need to be provided through multiple channels (health professionals, other family members, and community) and in several ways, such as monitoring vital signs and stress values through wearable devices to establish an early warning of the risk of negative emotions; that timely physical or pharmacological treatments can be administered to enhance health behaviors and motivation.

Another essential finding of this study was that most older adult stroke patients were confused about searching for health information and were skeptical about its accuracy during rehabilitation. Accurate health information search through multiple pathways is highly effective in treatment selection, symptom management, prognosis, survival, coping skills, doctor-patient communication, and conscious decision-making (Sedrak et al., 2020; Link and Baumann, 2023). Subjective behaviors and attitudes influence health information search and judgment (Lee et al., 2014). High-quality search pathways can reduce patient confusion and skepticism about health information. Stroke survivors would benefit from establishing online support groups that provide multiple avenues for community or social resources. For example, patients can readily access hemiplegic limb movement videos, knowledge about taking medications such as statins or learning about diseases such as pressure ulcer prevention through specialized online platforms. During hospitalization, survivors and caregivers must be assessed for their stroke-related knowledge and skills in accessing online resources, with health education and training to improve search skills. Patients are encouraged to seek information directly from health information providers in various ways, such as by phone or text message, and evidence-based comparative methods are used to promote consistent information-seeking active participation in shared decision-making and disease self-efficacy. After discharge, a nurse-led case manager model (Lin et al., 2022) can be tried to ensure that patients are given adequate health resource support while reducing nursing pressure.

In addition, survivors’ experiences have shown that the older population’s current health information search system could be better, with cumbersome search pages and complex medical terminology. The correct understanding of information affects the practical search for health information among older adults (Link et al., 2022), who generally do not have a high level of education and have difficulty understanding and finding complex information. It is recommended that the government promote research on natural language processing in the health field, build a language dictionary related to medical terms to improve the accuracy of matching health information search results, and promote natural human-computer interaction in the health field, such as searching for disease and health information through voice, pictures, or videos, to ensure that the survivors can quickly and correctly understand the health information and familiarize themselves with their diseases in the process of disease management, and ultimately to reduce the Disease Fear.

## 5. Limitations

This study had several limitations. First, throughout the study, there was no specific investigation of how caregivers’ search experiences impacted survivors’ health behaviors (e.g., as a spouse or child of the patient). Future studies should consider exploring this aspect further. Second, participant recruitment was limited to a relatively small geographic area and to stroke survivors who had experience searching for health information online which may reduce the generalizability of the findings. Additionally, because survivors’ needs for different types of rehabilitative care and disease information vary depending on the time since stroke, this is another factor that could be considered in future studies to better match survivors with specific services needed.

## 6. Conclusion

Our study provides insights into the current experiences and needs of older Chinese ischemic stroke survivors regarding digital health information search, focusing on impediments in the search process. Although survivors revealed they could access a wide range of health information *via* the internet, efficient access to and content of specialized health information emerged as the most popular suggestion to address a wide range of survivor needs. Future research may investigate survivors’ longitudinal experiences of the health information search process over an extended period to identify service components that need to be improved.

## Data availability statement

The original contributions presented in the study are included in the article/supplementary material, further inquiries can be directed to the corresponding author.

## Ethics statement

This study was reviewed and approved by the Investigation and Behavioral Research Ethics Committee of Nanjing Drum Tower Hospital, the Affiliated Hospital of Nanjing University Medical School (2022-692). Following the Declaration of Helsinki, the rights and security of the participants were guaranteed. During the recruitment process, study objectives and procedures were explained to participants, and written informed consent was obtained.

## Author contributions

LC, XQ, FW, and CJ: conceptualization. XQ and YH: methodology. YH and LH: formal analysis and writing—original draft. LH and MH: resources. MH and YH: revising and editing. YH, XQ, CJ, FW, MH, LH, and LC: writing—review and editing, investigation. All authors contributed to the article and approved the submitted version.

## Funding

This study was supported by the Special Fund for Clinical Research of Drum Tower Hospital, School of Medicine, Nanjing University (grant no. 2021-LCYJ-MS-05) and the Innovation Program for Postgraduate Research Practice of Jiangsu Province (grant no. SJCX23_0825).

## Conflict of interest

The authors declare that the research was conducted in the absence of any commercial or financial relationships that could be construed as a potential conflict of interest.

## Publisher’s note

All claims expressed in this article are solely those of the authors and do not necessarily represent those of their affiliated organizations, or those of the publisher, the editors and the reviewers. Any product that may be evaluated in this article, or claim that may be made by its manufacturer, is not guaranteed or endorsed by the publisher.
